# Transgenerational Effects and Epigenetic Memory in the Clonal Plant *Trifolium repens*

**DOI:** 10.3389/fpls.2018.01677

**Published:** 2018-11-20

**Authors:** Alejandra Pilar Rendina González, Veronica Preite, Koen J. F. Verhoeven, Vít Latzel

**Affiliations:** ^1^Institute of Botany of the Czech Academy of Sciences, Průhonice, Czechia; ^2^Molecular Genetics and Physiology of Plants, Ruhr-Universität Bochum, Bochum, Germany; ^3^Department of Terrestrial Ecology, Netherlands Institute of Ecology (NIOO-KNAW), Wageningen, Netherlands

**Keywords:** clonal reproduction, *T. repens*, plant memory, abiotic stress, transgenerational effects

## Abstract

Transgenerational effects (TGE) can modify phenotypes of offspring generations playing thus a potentially important role in ecology and evolution of many plant species. These effects have been studied mostly across generations of sexually reproducing species. A substantial proportion of plant species are however reproducing asexually, for instance via clonal growth. TGE are thought to be enabled by heritable epigenetic modification of DNA, although unambiguous evidence is still scarce. On the clonal herb white clover (*Trifolium repens*), we tested the generality of clonal TGE across five genotypes and five parental environments including soil contamination and above-ground competition. Moreover, by genome wide-methylation variation analysis we explored the role of drought, one of the parental environments that triggered the strongest TGE. We tested the induction of epigenetic changes in offspring generations using several intensities and durations of drought stress. We found that TGE of different environments were highly genotype specific and all tested environments triggered TGE at least in some genotypes. In addition, parental drought stresses triggered epigenetic change in *T. repens* and most of the induced epigenetic change was maintained across several clonal offspring generations. We conclude that TGE are common and genotype specific in clonal plant *T. repens* and potentially under epigenetic control.

## Introduction

Adaptive phenotypic plasticity allows plants to adjust their morphology to actual environmental conditions in order to maintain or increase their relative fitness. However, plant phenotype can also reflect past environments of parents or even grandparents (e.g., Miao et al., 1991; Latzel et al., 2014; Lampei et al., 2017) due to transgenerational effects (TGE). TGE had been studied mostly across sexual generations and only rarely among clonal generations (e.g., Raj et al., 2011; Verhoeven and van Gurp, 2012; Rendina González et al., 2016, 2017). Nonetheless, clonal reproduction is very common reproductive strategy in many plant communities and is often the main reproductive strategy for most plant species. For example, up to 70% of central European meadow species reproduce clonally (Klimeš et al., 1997). Clonal plants usually also exhibit complex and sophisticated behavior such as active foraging for resources (Bell, 1984; Waters and Watson, 2015) or division of labor (Alpert and Stuefer, 1997) where individual ramets might be adjusted to different functions like soil resources acquisition vs. photosynthesis. In this regard, each new ramet can be considered as a new generation potentially independent from the main mother plant carrying on environmental information (Latzel et al., 2016). It has been recently shown that the behavior of clonal plants can be governed not only by actual environmental condition but also by their past experiences, i.e., by TGE (Louapre et al., 2012; Rendina González et al., 2016, 2017). It is evident that TGE in clonal plants should get more attention in order to improve our understanding of their role in ecology and evolution.

TGE can be a simple consequence of carry over effects when chemicals and/or pathogens are passed from parents to offspring (Roach and Wulff, 1987; Rossiter, 1996; Huxman et al., 2001). Although, more often, TGE are thought to be targeted by a pre-programming of offspring phenotypes likely via *epigenetic mechanisms* (Bruce et al., 2007; Ginsburg and Jablonka, 2009; Ding et al., 2012; Thellier and Lüttge, 2013; Müller-Xing et al., 2014). Epigenetic mechanisms comprise histone modification, methylation of cytosine residues of DNA and small RNA molecules regulating gene expression, which are intimately interconnected (Wagner, 2003; Vanyushin, 2006). DNA methylation is shown to be environmentally inducible and, in some cases, heritable (Boyko et al., 2010; Angers et al., 2010; Hauser et al., 2011; Wibowo et al., 2016; Lämke and Bäurle, 2017). Nonetheless, most of the environmentally induced epigenetic changes are maintained within generations and rarely passed to sexually derived offspring due to meiosis that resets most of the environmentally induced epigenetic variation (Feng and Jacobsen, 2011; Paszkowski and Grossniklaus, 2011; Heard and Martienssen, 2014; Tricker, 2015). Clonal plants, on the other hand, can reproduce asexually and thus bypass meiosis. Therefore, it has been proposed that environmentally induced epigenetic change can be better maintained in clonal than sexual generations. Heritable environmentally induced epigenetic change can consequently enable a rapid adaptation to changing environments and its implications in short-term microevolution of clonal plants (Latzel and Klimešová, 2010; Verhoeven and Preite, 2014; Dodd and Douhovnikoff, 2016).

Indeed, mounting evidence showed that epigenetic differentiation of clonal plant populations can be at least partly caused by environmental induction. One of the first evidences provided Verhoeven et al. (2010) who showed that environmental stress in parental generation can trigger changes in DNA methylation that can be passed to next apomictic (clonal) generation of dandelions (*Taraxacum officinale*) with high fidelity. The environmental induction of DNA methylation changes was genotype-specific and represents, at least partly, a stress-induced increase of seemingly untargeted DNA methylation variation (Preite et al., 2018). In another study, they pointed out that the epigenetic differentiation (DNA methylation variation) of natural populations of apomictic dandelions can be environmentally determined (Preite et al., 2015). Furthermore, Richards et al. (2012) showed in Japanese knotweed and Gao et al. (2010) in alligator weed that genetically uniform populations of clonal plants can be epigenetically structured, and that this structure is likely due to environmental conditions. Raj et al. (2011) observed in clonal offspring of poplar trees that drought stress response was associated with origin of a genotype and was likely mediated by epigenetic variation. Finally, Robertson et al. (2017) identified specific epigenetic variation in clonal *Spartina alternifolia* populations related to water pollution. Although these pioneering studies provided first evidence that epigenetic change can be triggered by environment, they did not detect direct phenotypic effects of epigenetic variation (e.g., Verhoeven et al., 2010) and were not able to distinguish between epigenetic variation originated from environmental induction or selection of certain epigenotypes (e.g., Richards et al., 2012; Spens and Douhovnikoff, 2016; Robertson et al., 2017).

Here we provide results of two experiments focusing on clonal TGE and epigenetic changes in *Trifolium repens* induced by various environments. In the first experiment, we tested the effect of five parental environments – control, drought, contaminated soil (salt and copper), and shading, on the induction of clonal transgenerational phenotypic effects in the common clonal herb *T. repens*. Since TGE can be genotype specific (Groot et al., 2017; Lampei et al., 2017; Münzbergová and Hadincová, 2017), we tested the generality of environmentally induced TGE of the five environments across five genotypes. Since drought stress triggered the strongest phenotypic TGE in one of the tested genotypes, we focused in the second experiment on drought stress for this single genotype only. By methylation-sensitive amplification polymorphism (MSAP), we tested the role of different intensities and durations of drought periods on induction of epigenetic change in clonal offspring generations. We tested the following hypotheses: (i) environmental stress experienced by the parental clone triggers clonal TGE observable at the phenotype level of clonal offspring ramets, (ii) clonal TGE are genotype specific, (iii) different intensity and duration of drought stress in the parental generation induces changes in DNA methylation that is passed to clonal generations.

## Materials and Methods

### First Study – Transgenerational Effects

Five genotypes of *Trifolium repens* L. were randomly collected from grasslands in surrounding of Průhonice town, Central Bohemia, Czech Republic in 2013. All genotypes thus experienced same climatic conditions and very similar (a)biotic interactions. Despite this, the genotypes differed in their growth where two of them produced more biomass than other three genotypes (see also below). Their propagation took place in a greenhouse with controlled temperature and light regime set up at 17 h light and 7 h dark. Plants were cultivated in 30 × 40 × 8 cm trays filled with commercial Agro lawn soil substrate (mixture of compost, peat and sand, same substrate was used in all steps of the study) for 4 months to even out possible environmental effects and to pre-cultivate plant material.

### Study Design

**FIGURE 1:**
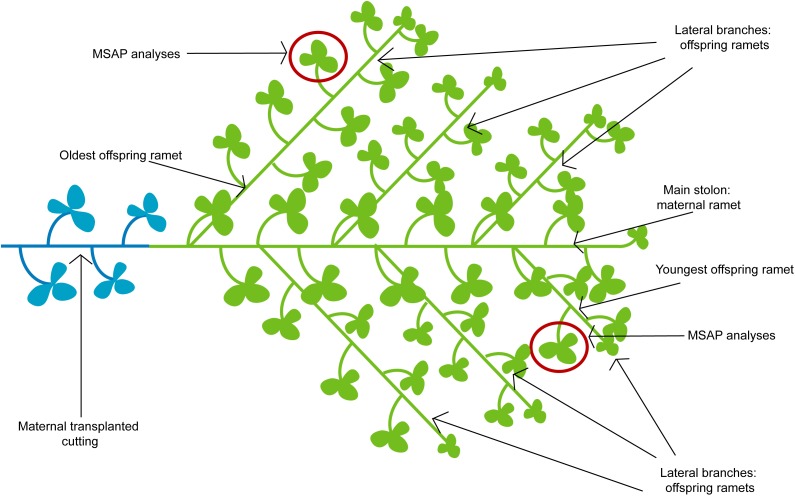
Idealized scheme of *T. repens* plant developed after transplantation of maternal cutting to a control environment. Highlighted are leaves collected for MSAP analyses in the second experiment and the cohorts of offspring ramets (i.e., the oldest and youngest offspring ramets).

Ten cuttings consisting of eight internodes of each genotype were individually transplanted to 30 × 40 × 5 cm trays filled with soil substrate and were let to propagate. After 1 month plants were proportionally and randomly assigned to the following environments for 2 months: control – no manipulation, drought – limited watering only when leaves were wilting (eight cycles of drought stress during the run of experiment), copper contaminated soil – regular application (three times in a week, 24 applications in total) of 60 ml of 16 mM solution of copper (II) sulfate pentahydrate (CuSO_4_ × 5H_2_O) with estimated final concentration of 500 mg copper in 1 kg soil, salt contaminated soil – regular application of 100 ml of 4.3 mM solution of salt (NaCl) with estimated final concentration 1.5 g salt in 1 kg soil, and shading by growing plants under green plastic sheet that reduced light intensity at 50%. After 2 months of cultivation in all environments, 10 cuttings (maternal ramets) consisting from 4 internodes and apical end labeled by a plastic ring were created from each treatment and genotype combination and individually transplanted to 20 × 30 × 4 cm trays (one cutting per tray) filled with soil substrate without any further manipulation. All ramets were without root system with only emerging root tip usually at the fourth internode. All trays were randomly distributed in the greenhouse, and their positions were changed four times during the cultivation period. This randomized design resulted in 250 plants (5 genotypes × 5 treatments × 10 plants) in total. After 50 days from transplantation all plants were harvested. Every harvested plant was cut at the position of the plastic ring (position of the apical end at the time of transplantation), and only the parts that had developed after transplantation were considered. Every plant was divided into the maternal stolon (the main axis of growth of the transplanted maternal ramet, see also Figure 1) and into lateral branches, here considered as the collection of daughters (offspring) ramets due to the monopodial growth of *T. repens* (i.e., maternal ramets is elongating in the main axis and producing offspring ramets via the lateral axillary buds). Maternal stolon as well as all offspring ramets were dried at 80°C for 24 h and weighed.

### Second Study – Drought Induced DNA Methylation Variation in Clonal Offspring

Morphological data as well as experimental design of the study has been already published (Rendina González et al., 2016) and, therefore, we provide here only a reduced description of the study. We used the same growing setup as in previous study. Thirty cuttings of pre-cultivated genotype of *T. repens* were individually planted to a tray (30 × 40 × 8 cm) filled with commercial Agro lawn soil substrate (mixture of compost, peat and sand). After 30 days, plants were randomly assigned (six trays per treatment) to each of the following five treatments: (1) Control; (2) Long-Intense drought; (3) Short-Intense drought; (4) Long-Medium drought; and (5) Short-medium drought. Plants assigned to control treatment were watered regularly to maintain the soil permanently moist. For the intense drought treatments, plants were watered with approximately 200 ml of water only when most leaves were wilting. Such stress occurred within 4 to 7 days. The Long-Intense drought treatment was applied for 4 months (December 2014 to March 2015), whereas the Short-Intense drought treatment was subjected to the control treatment of the first two of these months (December 2014 to January 2015) and to the water stress for the next 2 months (February to March 2015). The medium drought plants experienced half of the drought cycles experienced by plants in the intense drought treatments, which was achieved by applying water stress to them during alternating periods of water stress experienced by plants in the intense drought treatment. The Short-Medium drought and Long-Medium drought corresponded to those already described for the intense drought treatments (for more detailed information see Rendina González et al., 2016). In April 2015, all drought treatments were terminated, and plants were cultivated for another 14 days in control conditions with a saturated water regime. After the period, five standardized apical cuttings from each parental plant from all treatments were created. These cuttings consisted of four nodes and the apical end and were planted individually into 18 × 10 × 6 cm trays filled with standard potting soil, i.e., one cutting per tray. Fifteen replicated plants from each parental treatment, 75 plants altogether, were randomized and grown for 2 months in control treatment conditions. After 2 months above-ground biomass was harvested (results published in Rendina González et al., 2016). Leaf samples for MSAP analyses were collected from five randomly selected plants from all treatments. Collected were fully developed leaf of the youngest and oldest offspring ramet that had developed after transplantation to the control environment (Figure 1). Generally, around five lateral branches, corresponding to five consequent generations, developed in our experiments. Together were collected 50 leaf samples (5 treatments × 5 replicated plants × 2 leaves). However, in total, only 41 samples from the offspring ramets were used for molecular analyses due to insufficient size for DNA extraction of 6 leaf samples from the Short-Medium drought treatment and 3 not properly amplified samples (2 from Short-Intense and 1 from Long-Intense treatments).

### MSAP Analysis

Total genomic DNA was extracted from 8 mg of dry leaf material with NucleoSpin^®^ Plant II kit (MACHEREY-NAGEL GmbH & Co. KG, Düren, Germany), and the quality was examined by electrophoresis in agarose gel 1% (w/v). Purity and quantification were measured spectrophotometrically with NanoDrop2000 (Thermo Scientific). DNA digestion was performed with 100 ng of DNA, 8 units of *HpaII* (New England) as frequent cutter and 8 units of *EcoRI* (New England) as rare cutter. The digested ends were ligated with 0.5 μl of *HpaII* adapter [50 μM] and 0.5 μl of *EcoRI* adapter [10 μM] (Table 1) and 4 units of T4 DNA ligase (New England). All samples were then diluted 6.66× fold.

**Table 1:** Results of GLM testing the effect of parental treatments and genotype on the biomass of maternal ramets of *T. repens* after transplantation to the control environment.

	Df	SS	*F*	*P*
Genotype (G)	4	1.714	29.808	**<0.0001**
Treatment (T)	4	0.889	15.463	**<0.0001**
G × T	16	0.331	1.437	0.128


*Significant P values are in bold face.*

*HpaII* recognizes CCGG sequences but cuts only if the cytosines are unmethylated or if the external cytosine is hemimethylated. Cleavage is blocked if the cytosine is fully methylated. Thus, in clonal plants, assuming the absence of genetic variation, MSAP loci can be interpreted as a direct variation in the methylation status of the restriction site (Verhoeven et al., 2010). MSAP protocol was adapted from Aina et al. (2004). A pre-amplification step was carried out with *HpaII* primer (5^′^GACTGCGTACCAATTC) and *EcoRI* with one selective nucleotide (5^′^GACTGCGTACCAATTC+A). PCR mix contained: 2 μl of diluted DNA; 2 μl Buffer 10× (TS); 1 μl ClMg^+2^, 0.8 μl dNTPs [5 μM], 0.4 μl. H-M primer [10 μM], 0.4 μl EcoR1 primer [10 μM]; 1 unit of *Taq* DNA Polymerase (ThermoScientific) in a final volume of 20 μl. The amplification conditions were: 94°C for 2 min; 10 cycles at 94°C 30 s, touchdown of -1°C 65°C for 30 s, 72°C for 2 min, 25 cycles at 94°C for 30 s, 56°C for 30 s, 72°C for 2 min and a final elongation step at 60°C for 10 min. The samples were then examined by electrophoresis in agarose gel 1% (w/v).

A second step of selective amplification was conducted with three fluorescently labeled primer pairs chosen from a preliminary selection test of 12 primers and 5 μl of the pre-amplificated DNA 10×, 2.5 μl Buffer 10× (TS); 1.5 μl ClMg^+2^, 1 μl dNTPs [5 μM], 0.5 μl BSA (TS), 0.5 μl HpaII selective primer [10 μM], 0.5 μl EcoR1 selective primer [10 μM]; 1 unit of *Taq* DNA Polymerase (ThermoScientific) in a final volume of 20 μl. The selective PCR conditions were as following: 94°C for 4 min 30 cycles at 94°C for 45 s, 60°C for 30 s, 72°C for 30 s and a final elongation step at 72°C for 5 min. The primer combinations used were EcoRI-AAG/HpaII-CAC, EcoRI-ATT/HpaII-TTA, EcoRI-ACA/HpaII-TCAA.

Following selective amplification, 1 μl of each the PCR products were mixed with a solution of 10 μl of Hi-Di formamide and 0.2 μl of molecular weight marker LIZ500 and denatured at 95°C for 5 min followed by quick cooling on ice.

### Fragment Analysis

The amplified fragments were separated by capillary electrophoresis in the ABI 3130 Genetic Analyzer (Applied Biosystems) and measured with the GeneScan^TM^-500 LIZ^®^ Size Standard (Applied Biosystems, Warrington, United Kingdom). Presence (1) and absence (0) of fragments were scored from the obtained electropherograms using GeneMarker 2.2.0 software (SoftGenetics^®^ LLC) to construct an epigenetic binary matrix. Fragments from approximately 100–500 bp were scored based on the presence of at least one peak height over 50 relative fluorescence unit and visually compared between all samples relative to each primer combination. Loci present in the negative controls and the ones which contained more than three miss-matches between technical replicates were removed from the analysis (26 in total). 10% of the samples were replicated to estimate the error rate together with the negative controls (Bonin et al., 2004). The error rate for all primer combinations was 6.7%.

### Statistical Analyses

#### First Study – Transgenerational Effects

The effects of maternal treatments and genotype on the dry biomass of maternal stolon and offspring ramets were tested using general linear model (GLM) with the two-way full factorial design. Since maternal stolon biomass had weak but positive effect on offspring biomass (correlation biomass of maternal ramet with offspring biomass *R*^2^ = 0.065), maternal stolon biomass was included as a covariate to the statistical model when offspring biomass was analyzed.

To meet the assumptions of homoscedasticity and normality, all measured variables were log transformed prior to analyses. All statistical analyses were performed using JMP statistical software (JMP 10, SAS Inst.).

#### Second Study – DNA Methylation Variation

For the analysis of the binary matrix, the “msap” package for R was used (Pérez-Figueroa, 2013). Population differentiation was tested using analyses of molecular variance (AMOVA) that estimates Phi-st as fixation index (an analog of Fst for molecular data, Excoffier et al., 1992) by means of the package “pegas” with 10,000 permutations and includes the package “ade4” for the principal coordinates analysis (Pérez-Figueroa, 2013). In total, all offspring ramets that had developed after transplantation of the maternal ramets to the control environment were included in the analysis (*n* = 41).

## Results

### First Study – Transgenerational Effects

#### Maternal Stolon

**FIGURE 2:**
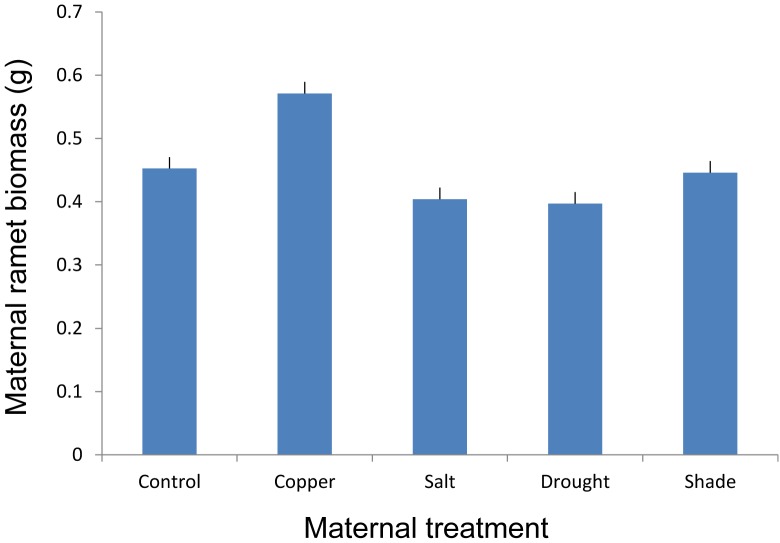
The effect of maternal treatments on the above-ground biomass of maternal ramets of *T. repens* that had developed after transplantation to the control environment. Means and SE are shown.

Maternal treatments altered growth of maternal stolon after its transplantation to the control environment (Figure 2 and Table 1). However, the *post hoc* test revealed that the significant difference was mainly due to copper treatment (Figure 2).

#### Offspring Ramets

**FIGURE 3:**
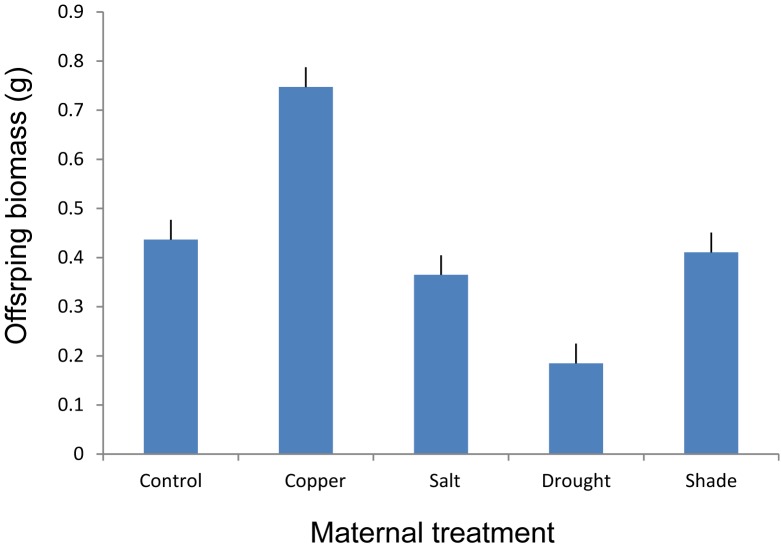
The effect of maternal treatments on the above-ground biomass of offspring ramets of *T. repens* that had developed after transplantation to the control environment. Means and SE are shown.

**FIGURE 4:**
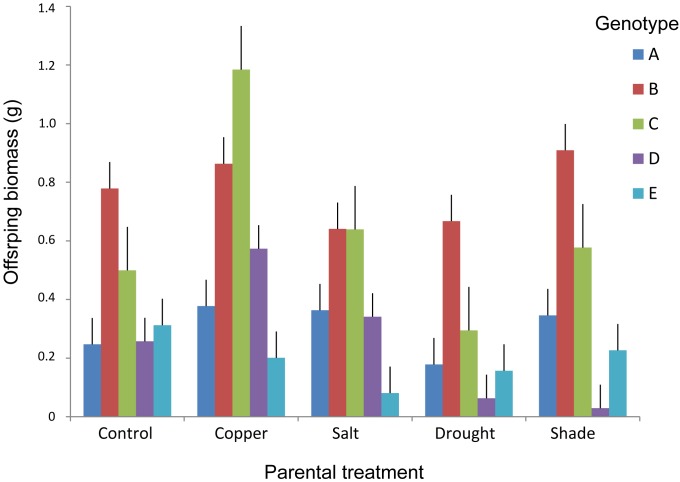
Above-ground biomass of offspring ramets of *T. repens* in relation to maternal treatments and genotypes. Means and SE are shown.

Maternal treatments affected biomass of offspring ramets, and this effect showed to be genotype specific (Table 2 and Figures3, 4). The offspring biomass was highest for the offspring of mothers that experienced copper contamination, the lowest biomass for the offspring of mothers that experienced drought stress (Table 2 and Figures2, 3). Nonetheless these effects were strongly genotype dependent (Table 2 and Figure 4). Copper treatment increased ramet biomass in two genotypes (C and F) but had no effect on offspring biomass of other genotypes. Shading had no effect on offspring biomass in all but one genotype. Salt contamination either decreased or increased offspring biomass depending on the genotype (Figure 4).

**Table 2:** Results of GLM testing the effects of maternal treatments and genotypes on the biomass of offspring ramets of *T. repens* developed after transplantation of maternal ramets to the control environment.

	Df	SS	*F*	*P*
Maternal stolon	1	3.034	41.206	**<0.0001**
Genotype (G)	4	8.252	28.0186	**<0.0001**
Treatment (T)	4	2.352	7.9847	**<0.0001**
G × T	16	3.321	2.819	**0.0004**


*Significant P values are in bold face.*

#### MSAP Analysis

**FIGURE 5:**
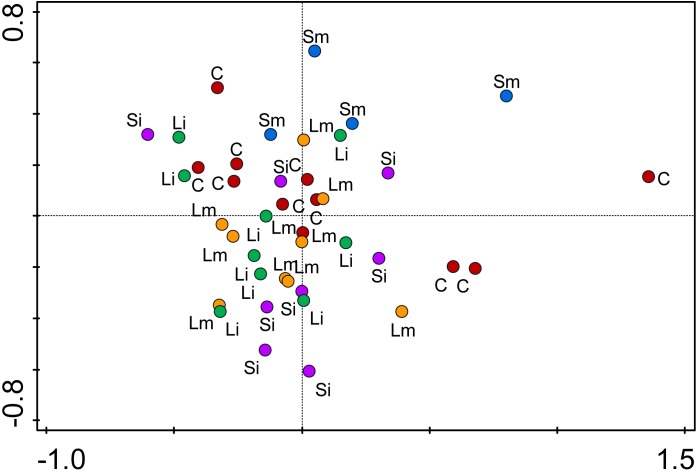
Principal Coordinate Analysis (PCoA) of epigenetic differentiation on offspring ramets of *T. repens* between treatments. C, control (red); Si, Short-Intense drought (purple); Sm, Short-medium drought (blue); Li, Long-Intense drought (green); Lm, Long-medium drought (yellow). The two coordinates explain 21% of the variance. Individuals are shown as points.

**FIGURE 6:**
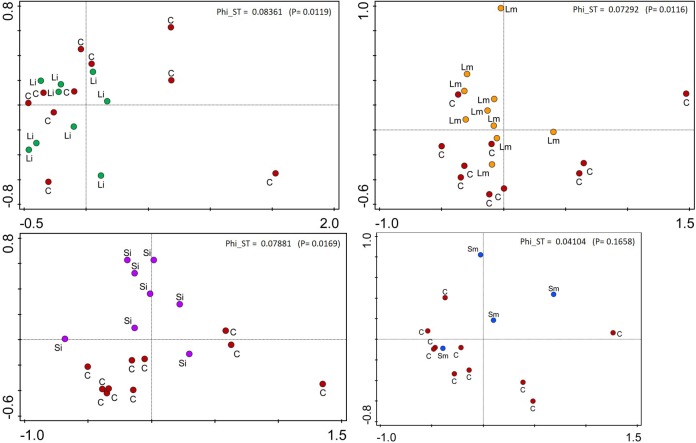
Representation of the PCoA on offspring ramets of *T. repens* for individual treatments vs. Controls. C, Control; Si, Short-Intense drought; Li, Long-Intense drought, Long-medium drought; Sm, Short-medium drought. Fixation index Phi_ST and *p*-value are shown for each plot.

AMOVA analysis of methylation profiles of leaf samples collected from the offspring ramets of all treatments that had developed after maternal ramets’ transplantation to the control environment showed a significant but low differentiation between treatment groups [Phi_ST = 0.07628 (*P* = 2e-04) see Figure 5]. Based on the epigenetic distances calculated from the binary matrix for the enzyme *HpaII*, two distinct groups formed in the PCoA with 21.8% of the variance explained in both axis (Figure 5). On the top the Short-medium differentiated from the rest of the treatments with the control treatment appearing in the middle of the plot, whereas the other three treatments (Short-intense, Long-intense, and Long medium) clustered together alongside the control treatment. Also, comparisons of individual treatments with controls show that epigenetic status of all treatments but short-medium drought differed from controls (Figure 6).

## Discussion

Transgenerational effects are thought to play an important role in the adaption and evolution of clonal plants in predictable fluctuating environments (Latzel and Klimešová, 2010; Verhoeven and Preite, 2014; Tricker, 2015; Bilichak and Kovalchuk, 2016). Our results indicate TGE positively or negatively altered clonal offspring biomass in *T. repens* depending on the type of maternal stress (drought, soil contaminations, and shading). However, the direction and strength of these TGEs were genotype specific. Finally, by testing the variation of genome-wide DNA methylation with MSAP, the second study shows that drought stress induces DNA methylation changes that are inherited in clonal offspring plants. Thus, DNA methylation has the potential to mediate at least some of the observed phenotypic TGE; however, the causal role of DNA methylation in the TGE remains to be demonstrated.

### First Study – Transgenerational Effects Due to Various Parental Stress Types

Copper contaminated soil in the maternal generation triggered a positive TGE on offspring biomass, whereas drought stress in maternal generation triggered a negative TGE on offspring biomass. Copper is an essential metal for plant growth and development since it is involved in a wide range of physiological processes, although it can be toxic at high concentrations (Sommer, 1931; Yruela, 2005). The threshold of copper, for which it becomes toxic to the plant is species dependent (Adrees et al., 2015). We observed a significant increase in the biomass production of maternal stolons and offspring ramets suggesting that the concentration applied in our study was probably below the toxic level for *T. repens*. Indeed, there are cases of copper tolerant plants that involve mechanisms to overcome the toxic effect of heavy metals. For example, the excess of copper can be sequestered into metabolically inactive parts like vacuole, apoplast, and epidermal cell walls (Adrees et al., 2015). This might be also the potential mechanism enabling phenotypic TGE due to the copper residual that can be transmitted from maternal plant to the clonal offspring, although TGE due to copper were found only in three out of five genotypes analyzed. Interestingly, despite that TGE were genotype specific, the copper treatment had similar effect on maternal ramets of different genotypes after their transplantation to control environment.

Repeated drought cycles in the maternal generation triggered the strongest TGE with a significant negative effect both on the maternal and offspring biomass. The most apparent mechanism enabling observed phenotypic TGE can be small size of maternal ramets due to their reduced growth in the dry maternal environment. Small size of maternal ramets can be translated into small size of offspring ramets due to reduced resources provided by maternal ramets. However, we controlled for this effect by including the size of maternal ramets as a covariate in statistical analyses. Even after accounting for the effect of maternal ramets’ size the results remained strongly significant suggesting that other mechanisms than the size of maternal ramets were also enabling TGE. These results are in line with our previous study (Rendina González et al., 2017) where we showed that despite drought-induced TGE significantly reduced clonal offspring biomass in optimal conditions, TGE were adaptive in the actual presence of drought, i.e., offspring ramets of mothers from dry environment performed better than offspring ramets of control mothers in the presence of drought. Another previous study (Rendina González et al., 2016) also suggested that TGE due to maternal drought were partly controlled by heritable DNA methylation change given that phenotypic TGE were not detected in plants that were treated with 5-azacytidne, a demethylating agent that removes epigenetic marks on DNA. Moreover, our second study (see below) also suggest that DNA methylation change can be at least partly responsible for observed TGE due to drought in maternal generation.

Because water availability is limited in salt contaminated soils, it is expected that salt stress should have similar physiological effects on plants as drought (Bartels and Sunkar, 2005; Uddin et al., 2016). Indeed, similarly to TGE due to drought, we found negative effect of TGE induced by salt stress on offspring biomass, although the effect was weaker than TGE triggered by drought. TGE due to salt can be adaptive as demonstrated Suter and Widmer (2013). They discovered an acquired salt tolerance in the offspring phenotype of stressed *Arabidopsis thaliana* plants, and this effect was strongest when both parental lines were stressed. The authors suggest that the observed TGE dependency on plant genotype can be explained by the interaction between the genetic background and the inheritance of environmentally induced epigenetic patterns (Suter and Widmer, 2013).

Of all stress treatments analyzed, shade did trigger TGE in one genotype of *T. repens* only. Previous studies have shown the role of light in fitness and memory of plants (Galloway and Etterson, 2007; Müller-Xing et al., 2014). For example, a study on an annual herb *Campanulastrum americanum* showed that different maternal light environments differently determine offspring germination rate and fitness (Galloway and Etterson, 2007). Since *T. repens* grows mostly in open biotopes such as grasslands or disturbed biotopes, it is likely that shade is not crucial stressor for the species and TGE due to shade were not evolutionary relevant.

Also, other studies have reported similar phenotypic responses of clonal offspring to drought or other biotic and abiotic stresses in clonal plants and model species (e.g., Verhoeven et al., 2010; Raj et al., 2011; Verhoeven and Preite, 2014; Herman and Sultan, 2016) and suggest that epigenetic mechanisms are likely involved (Wang et al., 2010; Zheng et al., 2013; Tang et al., 2014; Guarino et al., 2015; Douhovnikoff and Dodd, 2015; Ahn et al., 2017; Yaish, 2017).

Observed genotype specificity of most of TGE in our first study are in line with majority of other studies (Raj et al., 2011; Suter and Widmer, 2013; Latzel et al., 2014; Groot et al., 2017). Such specificity of TGE can have multiple origins. Each genotype can differ in its response to environmental stimuli, which can be translated into variation in TGE. In addition, genotypes likely differ in their selection history (although we collected the genotypes from very similar conditions) and thus also TGE could evolved differently in different genotypes. In this regard, the response of individual genotypes to various stresses can considerably differ highlighting thus the importance of considering the degree of genetic variation that is involved in phenotypic plasticity and its correlation with epigenetic variation and its inheritance. Various known molecular mechanisms are interconnected to give rise to the observed phenotype, and it is still not fully understood to what extent environmentally induced DNA methylation is independent from genetic control (Angers et al., 2010; Richards et al., 2012; Eichten et al., 2014). The interaction of hormones, stress responsive genes, small RNA involved in the RNA-directed DNA methylation pathway, and histone modifications are important players in the epigenetic landscape and its stability (Grant-Downton and Dickinson, 2006; Lauria and Rossi, 2011; Crisp et al., 2016). One mechanism playing part in epigenetic regulation is related to the activation and/or silencing of transposable elements. Some of these elements are thought to work dynamically when a genotype is challenged with stressful environments and are under strict epigenetic control (McClintock, 1984; Fedoroff, 2012). Thus, the mobility of transposable elements and changes in gene expression due to environmental cues might be heritable through DNA methylation that still retains a degree of reversibility (soft inheritance) in case of environmental fluctuations, which enables a stress “memory.” This soft inheritance might account even more in organisms reproducing clonally, avoiding the complex genetic shuffling that occurs during a meiotic event (Jablonka and Lamb, 2008; Latzel et al., 2016). Nevertheless, it remains an open question which molecular mechanisms are involved within each genotype and its differential reaction under same environmental conditions.

### Second Study – Methylation Profiles of Clonal Offspring

We found that the methylation profiles of offspring of all drough treatments (Long-intense, Long-medium, and Short-Intense) except the Short-medium drought stress, differentiated from controls (Figure 6). Morphological data on the same plants (Rendina González et al., 2016) also showed significant morphological differences between offspring of drought stressed mothers and controls. The role of DNA methylation in TGE was also indirectly supported by experimental demethylation of part of the plants in Rendina González et al. (2016), where the observed TGE disappeared after demethylation with 5-azacytidine under the same drought treatments. Although MSAP data do not provide insight in functional loci it shows DNA methylation differences. Our observations thus seem to be consistent with the idea that epigenetic variation was at least partly involved in observed TGE. However, there are other potential factors that can contribute to TGE like somatic transfer of hormones involved in response to drought (e.g., Roach and Wulff, 1987).

Our study adds to the mounting evidence that heritable epigenetic variation in plants can play a responsive role in the presence of stressful environments (Hauser et al., 2011). For instance, Herman and Sultan (2016) observed drought-triggered TGE in *Polygonum persicaria* that were removed after demethylation treatment with zebularine, thus indicating that DNA methylation was likely involved in the expression of the offspring phenotypes. On the other hand, Preite et al. (2018) observed a build-up of DNA methylation variation after three generations in two lineages of the apomictic dandelion concluding that these changes were inherited in a genotype and context-specific manner. Another study reported heritable DNA hypomethylation and enhance tolerance to heavy metal stress in the unstressed offspring of rice (Ou et al., 2012). Nevertheless, in order to gain a better insight into the heritability and stability of environmentally induced epigenetic modifications, it will be necessary to employ advanced methodological techniques into further studies questioning the role of environmental stresses in adaptation and evolution of epigenetic mechanisms in clonal plants.

## Conclusion

Overall, our results show that TGE are genotype specific in *T. repens* (and probably in other clonal plants too) and that there is potential for environment-induced, heritable DNA methylation changes to mediate TGEs. However, such DNA methylation based TGE probably exist in addition to other parental effects, such as carry over effects, e.g., copper residuals inherited somatically. This opens the question how behavior and/or ecology of clonal plants can be determined by their previous experiences (e.g., Latzel et al., 2016). Since clonal plants can exhibit very sophisticated behavior like foraging for resources, division of labor, or resources and information exchange among ramets, TGE can have strong potential to modify clonal plant behavior and thus their ecology and evolution. Nonetheless, to get more accurate overview of the role of TGE in ecology and evolution of clonal plants it would be necessary to test the adaptiveness of TGE (e.g., Rendina González et al., 2017) and importantly, the stability of TGE across several clonal generations and their overall generality across clonal species.

## Author Contributions

VL designed the experiments. VL and AG performed the experiments and statistical analyses. AG performed the molecular analysis. VP and KV aided in interpreting the results and worked on the manuscript. AG and VL wrote the manuscript with input of all authors.

## Conflict of Interest Statement

The authors declare that the research was conducted in the absence of any commercial or financial relationships that could be construed as a potential conflict of interest.
